# Autocrine interleukin-23 promotes self-renewal of CD133^+^ ovarian cancer stem-like cells

**DOI:** 10.18632/oncotarget.12579

**Published:** 2016-10-12

**Authors:** Dan Wang, Tong Xiang, Zhongquan Zhao, Kailong Lin, Pin Yin, Lupin Jiang, Zhiqing Liang, Bo Zhu

**Affiliations:** ^1^ Department of Obstetrics and Gynecology, Southwest Hospital, Third Military Medical University, Chongqing 400038, China; ^2^ Institute of Cancer, Xinqiao Hospital, Third Military Medical University, Chongqing 400037, China; ^3^ Department of Oncology, No. 421 Hospital of PLA, Guangzhou 510318, China; ^4^ Department of Oncology, Fuzhou General Hospital, Fuzhou, Fujian 350025, China

**Keywords:** cancer stem cells, IL-23, self-renewal, ovarian cancer

## Abstract

Cancer stem cells (CSCs) are a group of cells which possess the ability of self-renewing and unlimited proliferation. And these CSCs are thought to be the cause of metastasis, recurrence and resistance. Recent study has found that pro-inflammatory cytokine and chemotactic factor mediate the self-renewing and differentiation of most of CSCs. Thus we speculate that ovarian cancer stem cells (OCSCs) can also maintain the ability of self-renewing and differentiation by releasing inflammatory factor. This report we discuss the biological characteristics and the specific molecular mechanism mediated by interleukin-23 (IL-23) and its receptor on the self-renewing of OCSCs. We found that OCSCs had high expression of IL-23 and IL-23R. IL-23 could promote the self-renewal ability of OCSCs and played a very important role to maintain the stable expression of stem cell markers in vitro. Moreover, we verified that IL-23 could maintain the potential tumorigenic of OCSCs in vivo and mediate the self-renewal ability and the formation of tumor in OCSCs by activating the signal pathways of STAT3 and NF-κB. In addition, human low differentiation tissues showed overexpression of IL-23. And IL-23 positively correlated to the expression level of CD133, Nanog and Oct4. In conclusion, Our discoveries demonstrate that autocrine IL-23 contribute to ovarian cancer malignancy through promoting the self-renewal of CD133^+^ ovarian cancer stem-like cells, and this suggests that IL-23 and its signaling pathway might serve as therapeutic targets for the treatment of ovarian cancer.

## INTRODUCTION

Ovarian cancer is the leading cause of death among gynecological malignancy and the 5-year survival rate remains approximately 30% for the advanced-stage disease. About 70% of patients with ovarian cancer are diagnosed with advanced stages of the disease, and in the majority of the cases, they are characterized by chemotherapy resistance and widespread metastasis [1]. As a consequence, it is of great importance to understand the mechanisms that initiate and spread ovarian cancer.

Growing evidence disclose that a small fraction of ovarian cancer cells have potent tumor genesis capacities, which have been called cancer stem cells (CSCs) or cancer stem-like cells (CSLCs) [2, 3]. Recent research demonstrate that the presence of CSCs is one of the most pivotal factors that trigger ovarian cancer in a similar manner to several other cancers and this is based on their powerful self-renewing potential [4]. Furthermore, CSCs are confirmed to mediate tumor relapse and metastasis on account of their resistance to chemo- and radiotherapy [5]. Thus, further investigation on the biological attributes of CSCs may reveal a novel potential target for treatment of ovarian cancer.

CSCs exist in a special compartment called a CSC niche and this is thought to be beneficial to their biological function. It has been demonstrated previously that the microenvironment of the CSC niche is enriched with a broad spectrum of pro-inflammatory cytokines and chemokines. Heterogeneous populations of cells including infiltrating immune cells, stromal cells and endothelial cells in the CSC niche produce these cytokines and chemokines including IL-1, IL-6, IL-8 and IL-15 which mediate effects on the CSCs [6–9]. It is well-established that they provide paracrine signals to control the dynamic balance between self-renewal and differentiation of CSCs [10, 11]. Of note, emerging research found that an autocrine loop also exist in CSCs which directly influence their own function. Pro-inflammatory cytokines and chemokines such as IL-4, IL-6 and IL-8 can be secreted by CSCs to drive their self-renewal ability [12, 13]. However, the characteristics underlying the mechanism of regulation of CSCs in ovarian cancer remain to be explored.

IL-23 is a general pro-inflammatory cytokines that is mainly detected in activated macrophages, dendritic cells and keratinocytes in healthy skin [14]. It has a unique p19 subunit and a p40 subunit which is shared with IL-12 [15]. In our previous study, we found that OCSCs produced many cytokines including leukaemia inhibitory factor (LIF), IL-23, IL-1 and IL-8 and also high expressed many receptor molecule including IL-1R, IL-4R, IL-6R and IL-8R. Of which, OCSCs high expressing IL-23 and their receptor IL-23R caught our attention. There were some reasons as below: (1) Recent studies found that IL-23 participated in tumor growth and metastasis by directly binding to the IL-23 receptor that is expressed on cancer cells in multiple inflammation-associated cancers, including oral cancer, lung cancer, liver cancer and colorectal cancer [16–19]. Although IL-23 is involved in the progression of several types of tumors, its function in ovarian cancer is still unknown. (2) According to the previous literature reports, intestinal cancer could occur on the IL-23 transgenic mouse without the stimulation of carcinogen [20]. Due to the tumors incidence depended on the formation and self-renewal of the CSCs, so suggesting that IL-23 promoted self-renewal of CSCs may be one of the mechanisms of tumor formation. (3) In recent literature, it was reported that IL-23 had a significant rise in lung cancer tissues and the simulation from IL-23 in vitro could regulate lung cancer cell proliferation in two ways [17, 21]. Cell proliferation is the prerequisite and foundation for self renewal, so it indicates that IL-23/IL-23R signaling can also regulate CSCs. Moreover, this cellular behavior which is mediated by IL-23 is also characteristic of CSCs, and therefore the biological effects referring to the subpopulation of ovarian CSCs may be a novel underlying mechanism performed by IL-23 to mediate cancer initiation and progression.

Various pathways are involved in IL-23 downstream signaling transduction. The signal transducer and activator of transcription 3 (STAT3) and the transcription factor NF-κB are two major signaling pathways which are both pivotal signals to regulate CSCs self-renewal [22, 23]. In this study, we show that IL-23 and its receptor are both mainly expressed in ovarian CSC and IL-23 could promote its ability of self-renewal via activation of STAT3 and NF-κB signaling pathways.

## RESULTS

### CD133^+^ ovarian CSLCs express both IL-23 and its receptor IL-23R

Accumulating evidence suggests that CSCs, in the CSC niche, have complicated interaction networks with themselves or other adjacent cells which are mediated by a series of inflammatory cytokines and chemokines [12]. The essential premise on which this process is based is the active expression of related cytokines/chemokines and their related receptors. In order to figure out the regulation mechanisms of ovarian CSCs, we have performed a PCR array with respect to inflammation-associated genes in A2780-derived CD133^+^ ovarian CSLCs compared to CD133^−^ non-CSLCs. These cells have been confirmed to have CSCs characteristics including high capacity of self-renewal, tumorigenesis and multi-lineage differentiation properties as was demonstrated on our previous study [24, 25]. Of note, apart from upregulated cytokines which have been reported to take part in the biological processes of CSCs such as IL-1, IL-8 and IL-15 [6, 8, 9], we found another pro-inflammatory cytokine, IL-23, to also be upregulated in CSLCs compared to non-CSLCs (data not shown).

**Figure 1:**
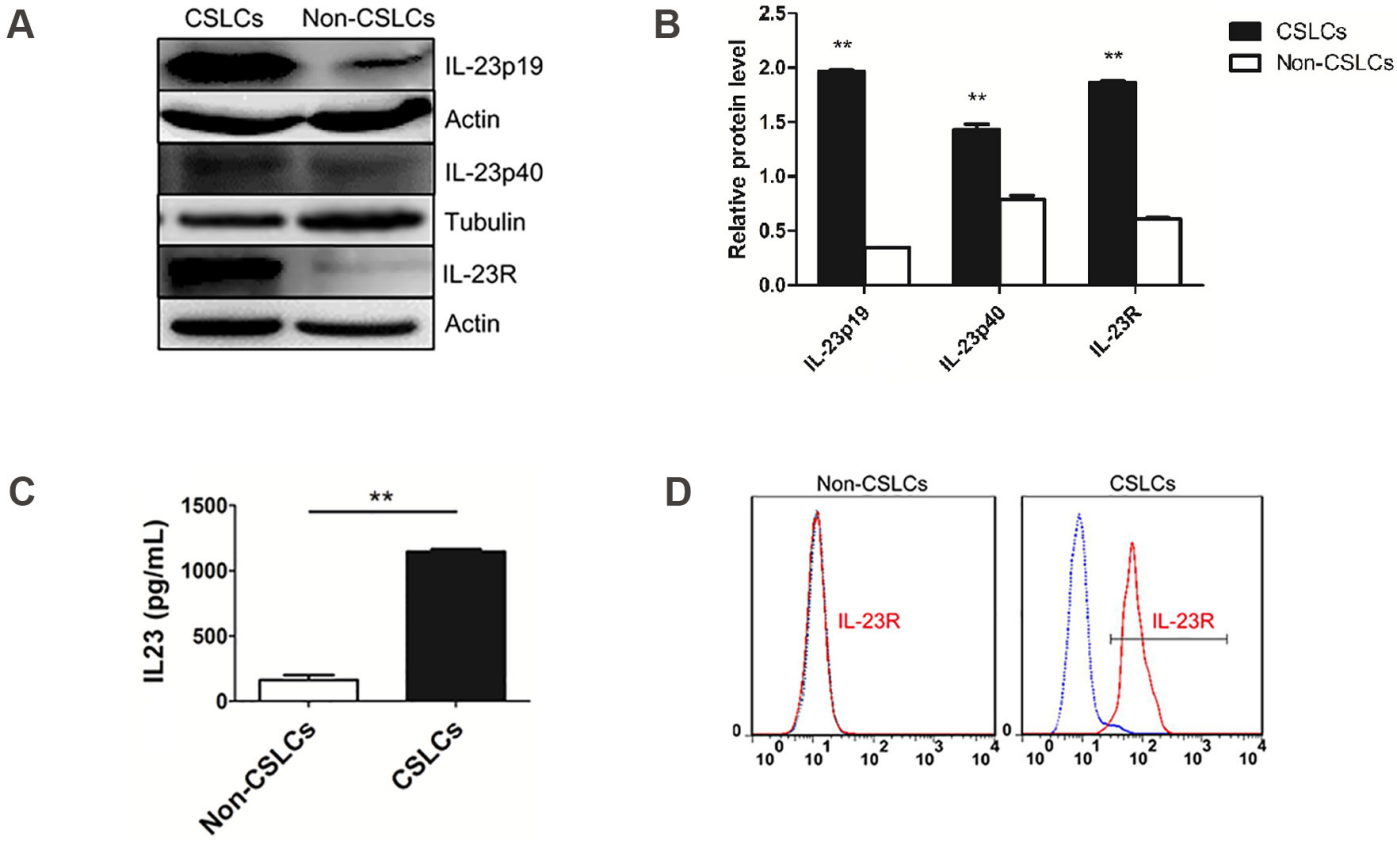
IL-23 and IL-23R expression in ovarian CSLCs **A.** Western blot analysis of levels of IL-23p19, IL-23p40 subunit and IL-23R in CD133^−^ and CD133^+^ cells, derived from A2780 cells. **B.** Quantification of expression data shown in (A). Data were expressed as as means ± S.D. **P < 0.01 vs. the CD133^−^ cells group, **C.** IL-23p19 protein expression levels, as measured by ELISA, showing an increase in IL-23p19 protein production by CD133^+^ cells relative to CD133^−^ cells. **D.** Flow cytometric analysis of IL-23R-expressing cells in A2780-derived CD133^+^ and CD133^−^ cells.

**Figure 2:**
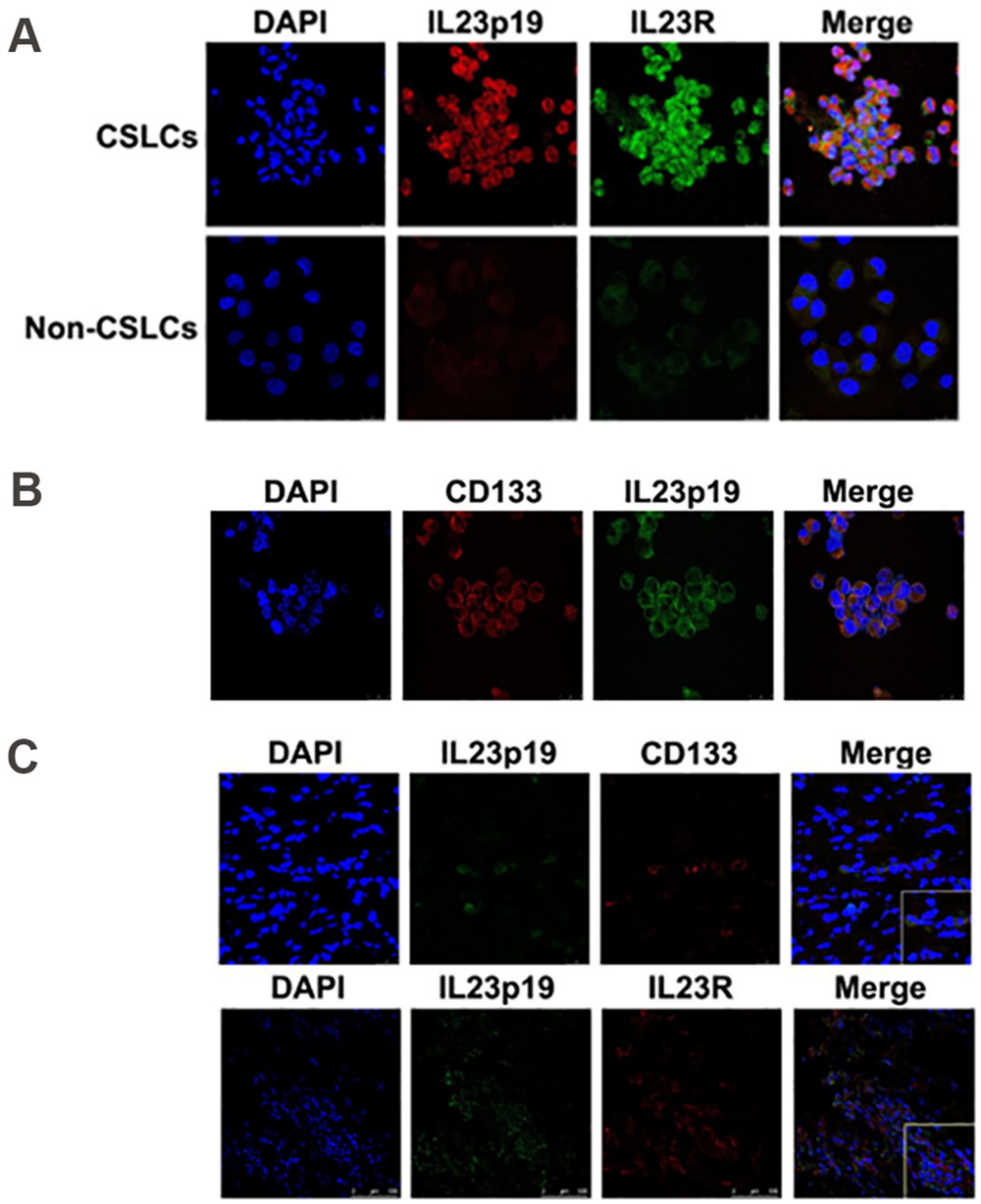
IL-23 and IL-23R expression in ovarian CSLCs **A.** Immunofluorescence detection of both IL-23p19 and IL-23R expression in A2780-derived CD133^+^ and CD133^−^ cells. **B.** Immunofluorescence detection of CD133 and IL-23p19 positive cells derived from A2780 cells. **C.** Immunofluorescence detection of IL-23p19 and CD133 positive as well as IL-23p19 and IL-23R positive cells isolated from primary ovarian cancer tissues. Scale bars, 25μm and 100μm.

To confirm this phenomenon, we use real-time PCR, ELISA, immunofluorescence and western blotting to investigate IL-23 expression level in A2780 and SKOV3-derived ovarian CSLCs and non-CSLCs. Consistent with PCR array results, both A2780 and SKOV3 CSLCs have significantly elevated IL-23p19 expression (Figures 1A-1C, Figure 2A, Supplementary Figures S1A-S1B, S1D). Furthermore, IL-23p19 is expressed at a higher level in CD133^+^ U87-derived glioma CSLCs (Supplementary Figure S1E). Coexpression of CD133 and IL-23p19 in CSLCs is shown in the Figure 2B. Additionally, the common subunit IL-23p40 is expressed in CSLCs demonstrating that an integral and active IL-23 molecule could be secreted by CSLCs (Figure 1A-1B). IL-23R expression was also measured by western blotting, immunofluorescence and flow cytometry. It is also upregulated in A2780 and SKOV3-derived CSLCs (Figure 1A, 1D, Figure 2A, 2C and Supplementary Figures S1C, S1D). To determine whether primary CD133+ CSLCs could also express IL-23 and IL-23R, we utilized immunofluorescence to investigate the expression of CD133, IL-23p19 and IL-23R on *in situ* ovarian cancer tissue samples. Both CD133/IL-23 and IL-23R/IL-23 are colocalized in primary tissue (Figure 2C). In addition, we isolated CD133^+^CSLCs from primary tissue using CD133 magnetic beads and IL-23R expression was found by flow cytometry. Taken together, we successfully proved IL-23 and its receptor were both highly expressed in CD133^+^ ovarian CSLCs and these data suggest that ovarian CSLCs may have an IL-23-associated autocrine pathway.

### Autocrine IL-23 promotes self-renewal of CD133^+^ ovarian CSLCs *in vitro*

Self-renewal is the leading attribute of CSCs which determines their major biological movements. The main signaling pathways of IL-23/IL-23R are STAT3 and NF-κB, which have shown to be involved in CSCs self-renewal [22, 23]. Thus it is possible that autocrine IL-23 may affect CSCs self-renewal through IL-23R and its downstream signaling. A sphere formation test was performed to measure self-renewal capacity of A2780 and SKOV3-derived CD133^+^CSLCs. Because IL-23 is highly expressed in CSLCs, we used three different methods to attenuate IL-23 levels and investigated the variation of sphere formation ability. A2780 and SKOV3-derived CD133^+^ CSLCs spheres were dissociated into single cells and were plated in 96-well plates at a density of 100 cells per well. Each intervention factor was applied, and after 5-7 days incubation, the number of newly formed spheres was counted.

**Figure 3:**
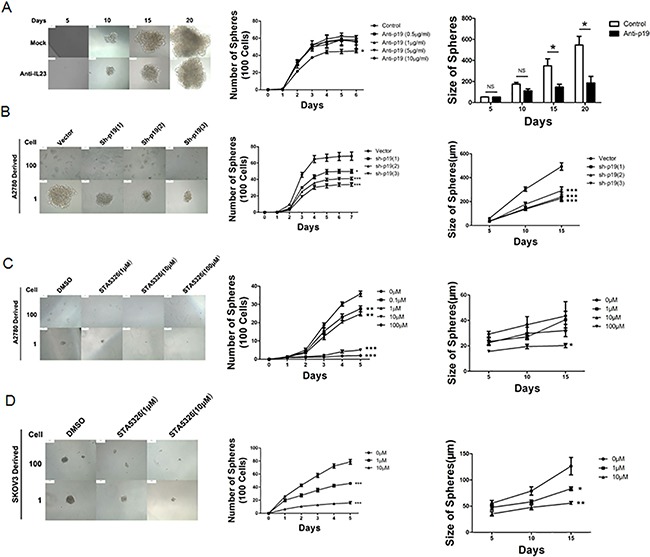
IL-23 is required for self-renewal capacity of CD133^+^ CSLCs **A.** A2780-derived CD133^+^ cells were dissociated into 100 and 1 cells and stimulated by different concentrations of IL-23p19 neutralizing antibody for 6 and 20 days. The number and size of spheres were measured. **B.** Control shRNA (vector) or IL-23p19-shRNA transfected CD133^+^ A2780 cells were dissociated in 100 and 1 cells and incubated for 7 and 15 days. The number and size of spheres were measured. **C, D.** A2780-derived (C) and SKOV3-dereved (D) CD133^+^ cells were dissociated into 100 and 1 cells and stimulated by different concentrations of IL-23 inhibitor STA-5326 for 5 and 15 days. The number and size of spheres were measured. All experiments were performed three times and data are expressed as mean ± SD. *P<0.05, **P<0.01, ***P<0.001.

Firstly, we added different concentrations of IL-23 neutralizing antibody (0.5, 1, 5 and 10μg/mL) and incubated cells for 6 days in order to block the function of soluble IL-23 in the environment. As shown in Figure 3A, the number of spheres significantly decreased in the presence of 10μg/mL. On the other hand, it has been reported that STA-5326, also known as Apilimod, in clinical application for patients with psoriasis, can effectively suppress synthesis of IL-12 and IL-23 in myeloid leukocytes at the transcriptional level [26, 27]. When CD133^+^ CSLCs were treated with STA-5326 with concentrations of 0-100μM for a 5-day incubation, from the second day, the number of CD133^+^ CSLCs spheres attenuated significantly in a dose dependent manner (Figures 3C-3D). In order to acquire a model whereby the IL-23 expression is stably decreased, we used lentivirus-mediated transfection. IL-23p19 interfering shRNA that carried green fluorescent protein (GFP) were transfected into A2780 and SKOV3-derived CD133^+^ CSLCs and this successfully downregulated the expression levels of IL-23p19 as measured by real-time PCR, western blotting and immunofluorescence (Supplementary Figure S2). Similar to the results of IL-23 neutralizing antibody and IL-23 inhibitor STA-5326, IL-23p19 interfering shRNA transduced CD133^+^ ovarian CSLCs presented decreased sphere formation capacity compared to which transduced with control vector carried with GFP (Figure 3B) The inhibition rate was approximately 30%-50% using 3 different target sequences of the IL-23p19 gene.

Sphere size can reflect the ability for stem cell self-renewal. Thus we dissociated A2780 and SKOV3-derived CSLCs spheres in a 96-well plate at the density of one cell per well. The dimension of the spheres that originate from a single cell was observed and measured at day 5, 10 and 15, and simultaneously, 3 methods to down-regulate IL-23 expression mentioned above were utilized. The size of spheres produced was reduced significantly in most cases (Figure 3).

Collectively, these data indicate that the self-renewal capacity of CD133^+^ ovarian CSLCs was predominantly mediated by IL-23 signaling in an autocrine manner *in vitro*.

### IL-23 plays an important role in the stable expression of stem cell markers of CD133^+^ ovarian CSLCs *in vitro*

**Figure 4:**
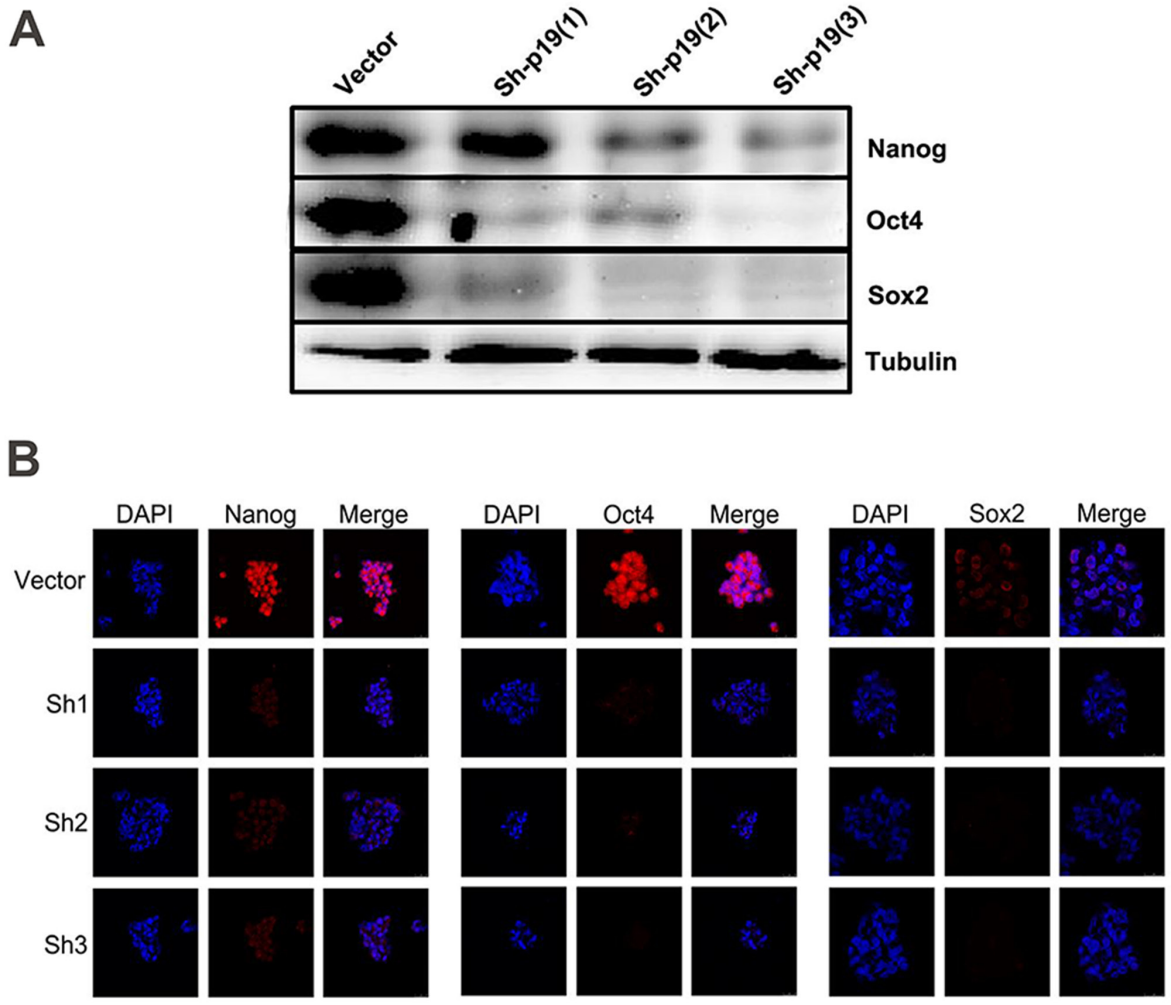
IL-23 is required for stable expression of stem cell markers in CD133+ CSLCs **A.** Representative western blot analysis of Nanog, Oct4 and Sox2 expression in Control shRNA (vector) or IL-23p19-shRNA transfected CD133^+^ A2780 cells. **B.** Immunofluorescence detection of Nanog, Oct4 and Sox2 expression in Control shRNA (vector) or IL-23p19-shRNA transfected CD133^+^ A2780 spheres. Scale bars, 25μm.

**Figure 5:**
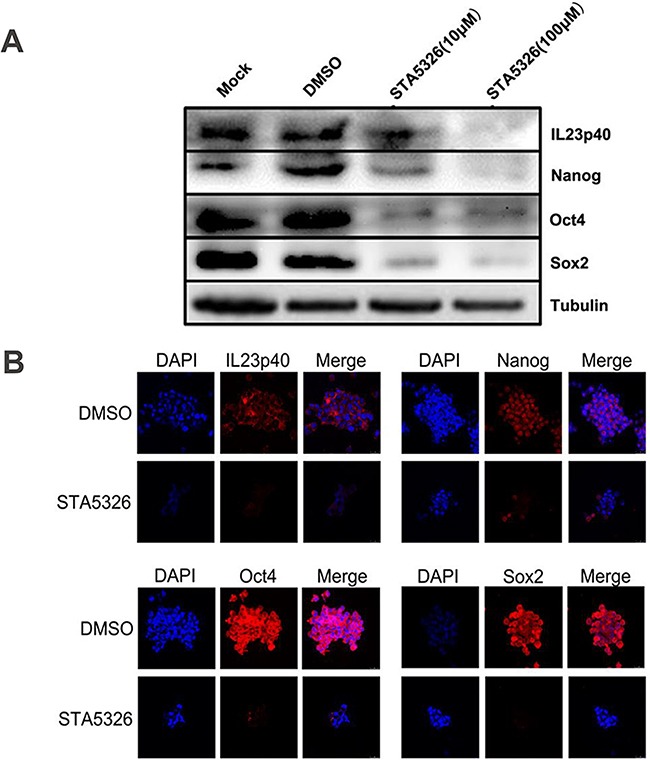
IL-23 is required for stable expression of stem cell markers in CD133+ CSLCs **A.** A2780-derived CD133^+^ spheres were pretreated with DMSO or different concentrations of STA-5326 for 48h or untreated (Mock). The protein extracts were subjected to western blot analysis. Representative results of Nanog, Oct4, Sox2 and IL-23p40 expression are shown in each group. **B.** A2780-derived CD133^+^ spheres were pretreated with DMSO or different concentrations of STA-5326 for 48h. Immunofluorescence detection was performed in each group. Scale bars, 25μm.

The transcription factors Nanog, Oct4 and Sox2 are three crucial stem cell markers required for efficient self-renewal of embryonic stem cells [28]. Emerging evidence has proved that these factors are widely expressed in many types of malignant tissues and display the hallmark CSC properties such as self-renewal, heterogeneous lineage differentiation and chemotherapy resistance [28–31]. On the basis of our results and well-established evidence that activation of IL-23/IL-23R and major downstream signaling pathways, STAT3 and NF-κB, have close associations with Nanog, Oct4 and Sox2 expression in embryonic stem cells and CSLCs [32–34], we hypothesized that autocrine IL-23 signaling may affect expression of these stem cell markers. To confirm this assumption, we pretreated A2780-derived CD133^+^ CSLCs with the IL-23 inhibitor, STA-5326. We also performed stable transfections of these cells with IL-23p19-shRNA to measure Nanog, Oct4 and Sox2 expression via western blotting and immunofluorescence. Surprisingly, in contrast to the control group, IL-23 knockdown through these two methods both had significantly lower expression of Nanog, Oct4 and Sox2 mRNA and protein (Figures 4A-4B, Figures 5A-5B). Moreover, the amount of downregulation of stem cell markers seen in the STA-5326 pretreated group was positively correlated with concentration of the drug used (Figure 5A). As a consequence, these results demonstrate that autocrine IL-23 signaling is consistent with the presence of stem cell markers being expressed in CD133^+^ ovarian CSLCs.

### Autocrine IL-23 maintains tumorigenic potential of CD133^+^ ovarian CSLCs *in vivo*

**Figure 6:**
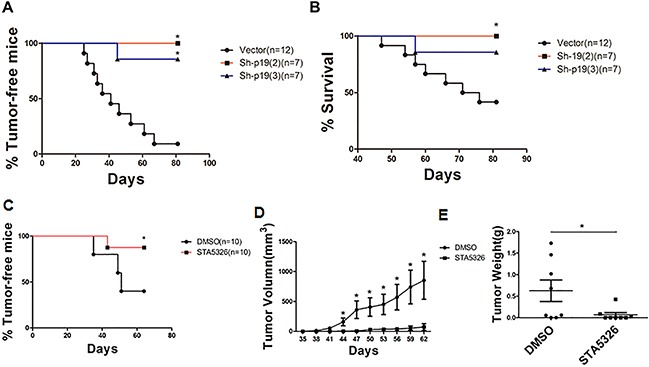
IL-23 mediates the growth of ovarian cancer xenografts *in vivo* **A, B.** A2780-derived CD133^+^ CSLCs (5 × 10^5^ per mouse) transfected with Control shRNA (vector) or IL-23p19-shRNA were injected subcutaneously into female nude mice and observed for 3 months before mice were killed. Tumor incidence (A) and survival rate (B) in each group are shown. **C-E.** A2780-derived CD133+ CSLCs (5 × 10^5^ per mouse) pretreated with IL-23 inhibitor ST-5326 (10μM) or DMSO for 48h were injected subcutaneously into female nude mice and observed for 2 months before mice were killed. Tumor incidence (C), xenografts tumor volumes (D) and weight of xenografts tumor (E) at day 62 are shown. Experiments were performed three times and data are expressed as mean ± SD. *P<0.05.

The importance of IL-23 signaling in the self-renewal of CD133^+^ ovarian CSLCs *in vitro* suggests that IL-23 might have an influence on tumorigenesis process of CSLCs. To further elucidate the function of IL-23 in CD133^+^ ovarian CSLCs self-renewal *in vivo*, we performed a xenograft experiment, in which IL-23p19 sh-RNA or control shRNA transfected A2780-derived CSLCs were injected subcutaneously into female nude mice of 6-8-weeks. The group of IL-23p19 knockdown mice with two different shRNA constructs showed significantly decreased tumor incidence and increased survival when compared to mice transfected with control shRNA (Figures 6A, 6B).

To further test this hypothesis, we pretreated A2780-derived CSLCs with IL-23 inhibitor STA-5326 (10μM) or DMSO for 48h and gave a subcutaneous injection to two groups of 6-8-week-old female nude mice [35]. When observed at 62 days, we found pretreatment with STA-5326 significantly reduced tumor incidence and the volume of resulting tumors were also reduced (Figures 6C, 6D). After the observation period, the weight of tumors in the group pretreated with STA-5326 was also significantly lower than those pretreated with DMSO (Figure 6E). In comparison to IL-23 knockdown, pretreatment of STA-5326 did not improve survival of mice bearing tumors (data not shown) suggesting the mode of delivery should be an important consideration for the drug to work effectively in mice.

These studies indicate that IL-23 is important in maintaining tumorigenic capacity of ovarian CSLCs and pharmacological targeting of IL-23 signaling may be beneficial for the treatment of ovarian cancer in a clinical setting.

### IL-23 signaling promotes CD133^+^ ovarian CSLCs through STAT3 and NF-κB activation

**Figure 7:**
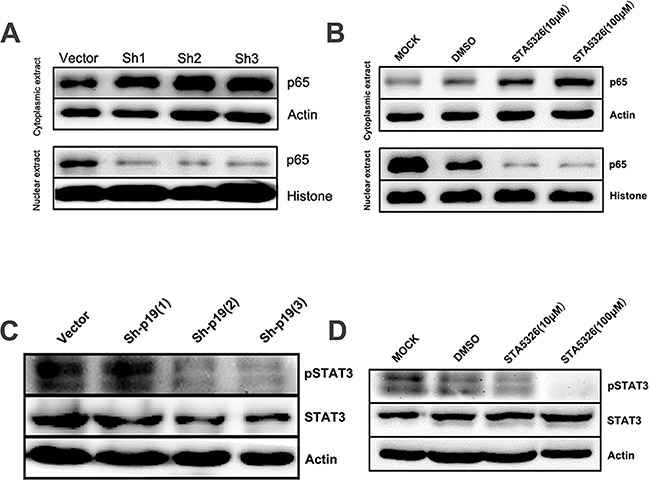
STAT3 and NF-κB signaling pathways are involved in IL-23-promoted self-renewal of CD133^+^ CSLCs **A.** Western blot analysis of p65 levels in cytoplasmic extract and nuclear extract from A2780-derived CD133^+^ CSLCs transfected with Control shRNA (vector) or IL-23p19-shRNA. **B.** Western blot analysis of p65 levels in cytoplasmic extract and nuclear extract from A2780-derived CD133^+^ CSLCs pretreated with DMSO or different concentrations of IL-23 inhibitor ST-5326 for 48h or untreated (Mock). **C.** Western blot analysis of p-STAT3 and STAT3 levels in total protein extracts from A2780-derived CD133^+^ CSLCs transfected with Control shRNA (vector) or IL-23p19-shRNA. **D.** Western blot analysis of p-STAT3 and STAT3 levels in total protein extract from A2780-derived CD133^+^ CSLCs pretreated with DMSO or different concentrations of IL-23 inhibitor STA-5326 for 48h or untreated (Mock).

As STAT3 and NF-κB are two major downstream mediators of IL-23 signaling and play important roles in CSCs [22, 23], we explored the manner by which activation of these two signaling pathway in A2780-derived CSLCs leads to modulation of IL-23 signaling. After 48h treatment with STA-5326, or IL-23p19 shRNA transfection, CSLCs display a significantly reduced level of phosphorylated STAT3 as demonstrated by western blotting (Figures 7C, 7D), and moreover, the degree of reduction is dependent on the concentration of STA-5326 (Figure 7D). Similar to the western blotting results of STAT3 in extracts from CSLCs, the active unit of NF-κB, p65, showed a reduced nuclear translocation as IL-23 expression is downregulated by STA-5326 or IL23p19-shRNA (Figures 7A, 7B). In addition, we also investigated the role of IL-23 in the activation of p38MAPK signal pathways. Result show that phosphorylation level of the P38 protein is no change in treatment group. (Supplementary Figure S5).

**Figure 8:**
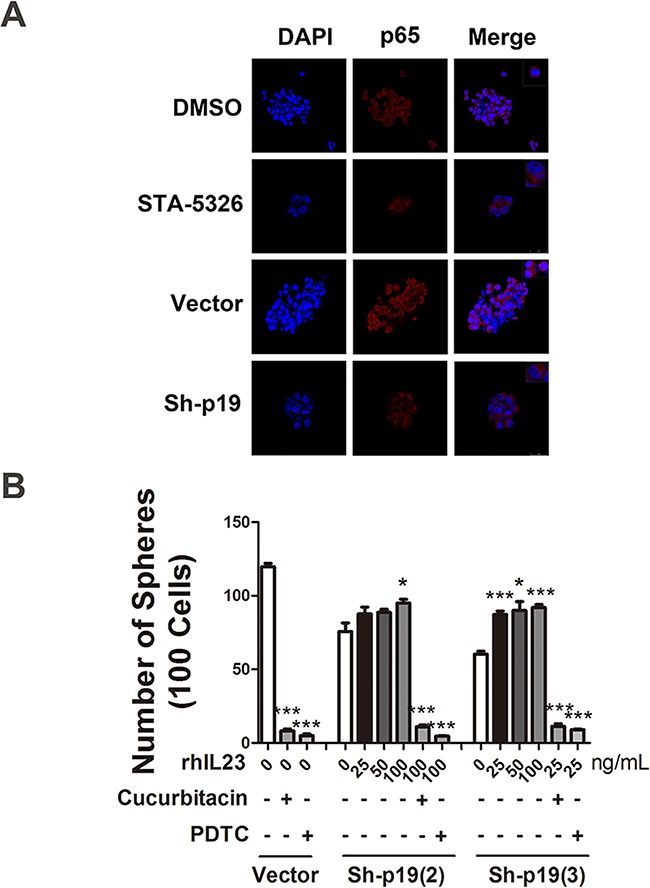
STAT3 and NF-κB signaling pathways are involved in IL-23-promoted self-renewal of CD133+ CSLCs **A.** Immunofluorescence detection of p65 nuclear translocation in A2780-derived spheres transfected with Control shRNA (vector) or IL-23p19-shRNA and pretreated with DMSO or IL-23 inhibitor ST-5326 (10μM) for 48h. The upper right corner of the figures present the cell morphology with enlarged images. **B.** A2780-derived CD133^+^ cells transfected with Control shRNA (vector) or IL-23p19-shRNA were dissociated into 100 cells and stimulated with different concentrations of recombinant human IL-23, STAT3 signaling pathway inhibitor Cucurbitacin and NF-κB signaling pathway inhibitor PDTC. Sphere numbers were counted in each group. All experiments were performed three times and data are expressed as mean ± SD. *P<0.05, **P<0.01, ***P<0.001.

Immunofluorescence staining results also display a reduction of NF-κB p65 nuclear enrichment when STA-5326 was added or the cells were transfected with IL23p19-shRNA (Figure 8A). Furthermore, we added different concentrations of recombinant human IL-23 (rhIL-23) (25, 50,100 ng/mL) accompanied with cucurbitacin (a STAT3 inhibitor) or PDTC (a NF-κB inhibitor) in a 96-well plate, plated with A2780-derived CD133^+^CSLCs which was transfected with IL23p19-shRNA, at a density of approximately 100 single cells per well, in order to observe their sphere formation number. At day 7, we found the number of spheres from cells with IL-23p19 knockdown partly restored in a dose-dependent manner when rhIL-23 was added and drastically decreased when cucurbitacin or PDTC was used at the same time (Figure 8B). A2780-derived non-CSLCs were also treated with rhIL-23 and IL-23 inhibitor STA5326 (100μM) and a CCK-8 proliferation assay was performed. No significant variation was found when rhIL-23 is added and STA-5326 showed a modest decline when read at OD450 (Supplementary Figure S3). Thus our results support an important role for IL-23-mediated STAT3 and NF-κB activation in CD133+ ovarian CSLCs self-renewal and tumorigenesis.

### IL-23 expression represents poor differentiation and positively correlated with CD133, Nanog and Oct4 expression in primary tissue

**Figure 9:**
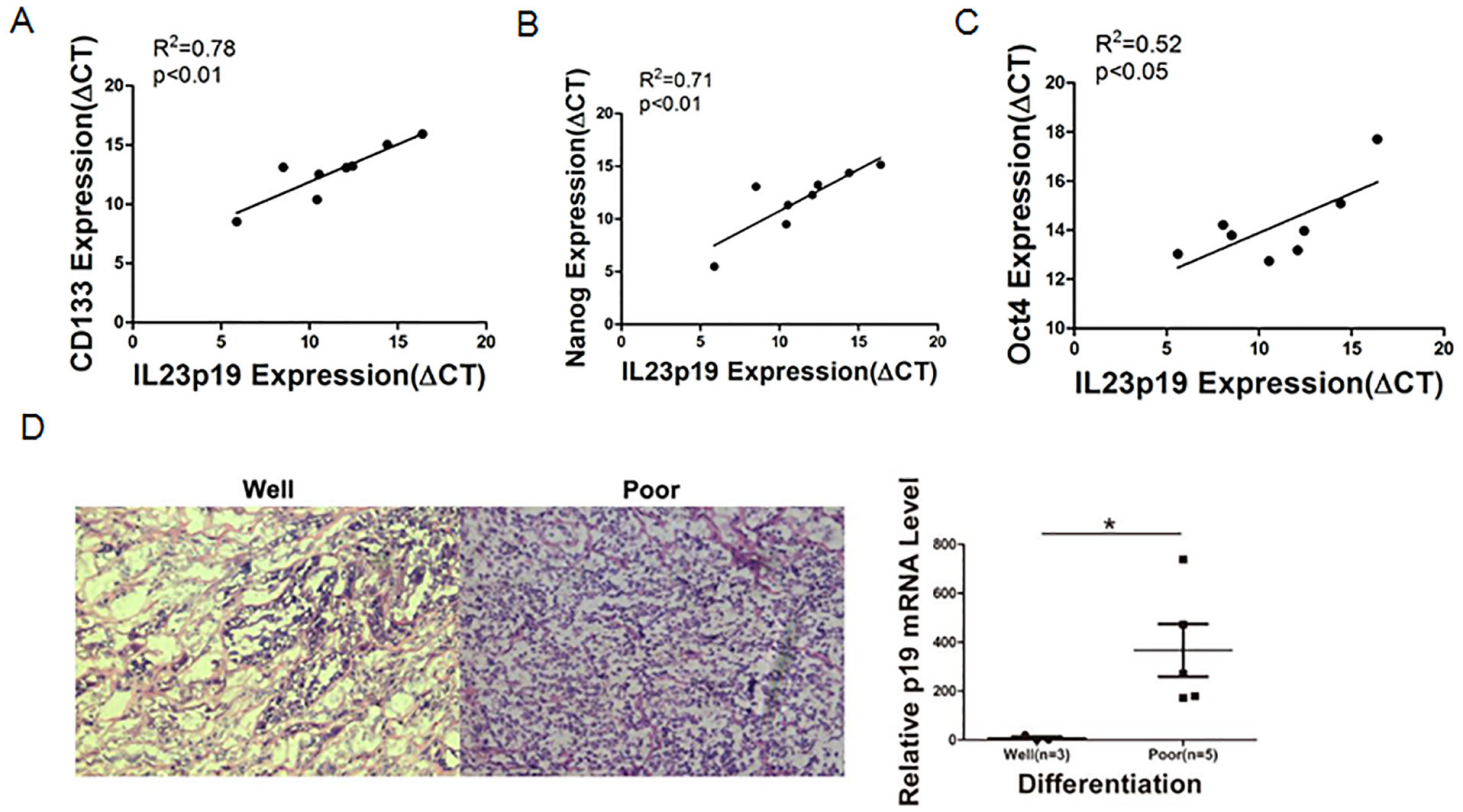
Correlation between IL-23p19 expression and CD133, Nanog, Oct4 levels and degrees of differentiation in primary ovarian cancer tissue **A-C.** Correlation of gene expression levels between IL-23p19 and CD133, Nanog and Oct4 in primary samples, as measured by qRT-PCRn. **D.** Representative H&E staining of well and poor degrees of differentiation in primary samples (left panel) and its relationship with IL-23p19 levels. Experiments were performed three times and data were expressed as mean ± SD. *P<0.05.

CSCs related markers are often differentiated poorly in clinical studies [36]. Our data support a close relationship between IL-23 and the crucial CSC markers. Therefore, we further used malignant tissue specimens from ovarian cancer patients to investigate the correlation between IL-23 expression levels and degrees of differentiation. The eight tumors used in this study from ovarian cancer patients were categorized as stage III serous adenocarcinomas. Then tumor specimens undergoing advanced ovarian cancer treatment were extracted and real-time PCR was performed to test IL-23p19 expression, and simultaneously, H&E staining was carried out to determine degrees of differentiation of the tissues. To our surprise, the specimen identified as low degree of differentiation had significantly higher IL-23p19 levels (Figure 9D). To further explore this phenomenon, we performed real-time PCR to investigate CD133, Nanog, Oct4 and Sox2 on these specimens. There are significant positive correlations between IL-23p19 and CD133, Nanog and Oct4 (Figures 9A-9C) but not Sox2 (data not shown). Thus we conclude that expression of IL-23 is positively correlated with CD133, Nanog and Oct4 levels in primary ovarian cancer tissue at the mRNA level and that higher IL-23 expression predicts poorer differentiation in histological grading.

## DISCUSSION

One of the emerging theories in tumor biology is that of CSCs and that these cells denote that the propagation and recurrence of cancers are administrated by a small subpopulation of cancer cells with strong proliferative and regenerative capacity [37]. In ovarian cancer, the CSC model has been postulated to drive tumor progression [2, 3, 38]. Numerous markers have been used to distinguish and characterize ovarian CSCs including CD133, CD44, CD117, epithelial cell adhesion molecule (EpCAM), CD24 and aldehyde dehydrogenase (ALDH) [2, 39–42]. In our previous study, we utilized CD133+ as the single cellular marker to isolate CSCs, and it appeared that CD133^+^ cells enriched from primary cancer tissues or derived from A2780 cell lines display high self-renewal capacity and tumorigenic potential [24, 25]. Thus CD133 was still used as an independent ovarian CSLCs marker in our study.

Emerging evidence shows that cancer progression is not simply an intrinsic cellular process that is triggered by accumulation of gene mutations in transformed cells. Tumor development also depends on the external signals existing in the tumor microenvironment. The biological behavior and functions of CSCs are also regulated by numerous signaling networks produced by surrounding cells or CSCs themselves [12]. Inflammation is the hallmark of cancer. Considerable clinical investigations have suggested a direct association between inflammatory states and cancer development. Several pro-inflammatory cytokines, such as IL-1, IL-6 and IL-8, participate in development of chronic inflammation have also been proven to induce and maintain procarcinogenic inflammatory microenvironment at the initiation of malignant transformation and tumor progression [43–45]. However, the influence of inflammatory cytokines on CSCs, especially ovarian CSCs, has been not fully elucidated. In order to determine the inflammatory states in ovarian CSC-niche, we observed the expression profile of inflammatory cytokines in CD133^+^ CSLCs derived from A2780 ovarian cancer cell line. Apart from previously reported cytokines, we found a cytokine that is not well-studied in ovarian cancer, IL-23, is highly upregulated. Interestingly, we also confirmed expression of IL-23R in CSLCs derived from cancer cell lines and primary cancer specimen. These lines of evidence all led us to focus on the role of IL-23 in regulation of CSLCs behavior.

IL-23 belongs to the IL-12 cytokine family which consists of IL-12, IL-23, IL-27 and IL-35 and these are heterodimeric cytokines formed from two subunits [46]. With respect to the physiological immune response, IL-23 is crucial for the development of Th17 cells and induces these cells to secrete IL-17 [47]. In addition, in a colorectal cancer model, it was found that gut microbial products induced IL-23 secretion from immune cells and further induced a protumoral IL-17 response [48]. Of note, IL-17 was also found to be an important cytokine that promotes self-renewal capacity of ovarian CSLCs as was reported by our previous study [26]. Thus the question to whether IL-23 could induce IL-17 secretion in CSLCs warrants future investigation.

A recent study demonstrated that exogenous IL-23 also had direct effects on proliferation of oral and pulmonary cancer cell lines by binding to the IL-23R expressed on them [16, 17]. In our study, only a weak expression of IL-23R is seen on A2780-derived non-CSLCs and neither rhIL-23 nor IL-23 inhibitor STA-5326 have any obvious influence on their proliferation capacity. Thus in an ovarian cancer model, the effect of IL-23 is not consistent with that in other types of cancer. However, surprisingly, based on a high expression of IL-23 and IL-23R in CD133^+^ ovarian CSLCs derived from cancer cell lines and primary cancer tissue, we found autocrine IL-23 could promote the self-renewal capacity and tumorigenic potential of CD133^+^ ovarian CSLCs both *in vitro* and *in vivo*. These results have shown a novel potential target of IL-23 associated with CSCs in ovarian cancer.

During the past years, a series of developmental pathways that regulate cancer stem cells have been elucidated. These pathways include Notch, Hedgehog, Wnt and human epidermal growth factor receptor 2 (HER2)-AKT [49]. The downstream signaling pathway of IL-23, STAT3 and NF-κB, are newly discovered pathways that control the biological behavior of CSCs such as its properties of self-renewal and cell survival [17, 20]. Here, we suggest STAT3 and NF-κB are involved in IL-23 mediated self-renewal of ovarian CSLCs. Of note, STAT3 is also the downstream signaling pathway of another crucial CSCs regulator IL-6 in glioma and colon CSCs [50, 51]. This fact may provide an indication that CSCs in different histological types of cancer have their own specific regulating signaling networks and IL-23 takes up the dominant parts governing regulation of ovarian CSCs. Conventional signaling pathways including STAT, NF-κB and Notch may be a common intracellular signaling axis in these networks. Taken together, our study emphasizes the important roles of STAT3 and NF-κB signaling pathways in ovarian cancer progression inferring to the ability of CSCs to undergo self-renewal.

The stem cell markers Nanog, Oct4 and Sox2 have also recently been proven to exert pivotal functions in CSCs-related oncogenesis [28–31]. However, the precise mechanisms of this process needs to be determined. A previous study showed that phosphorylated STAT3 binds to the murine Nanog promoter and activates its transcription [52–54]. These facts might help to construct a linkage between environmental signals and CSCs-related transcription factors. Thus it is likely that the variation of these stem cell markers in IL-23 induced signaling activation also depends on conventional intracellular signaling pathways such as STAT3. This is an ongoing research to confirm and improve our current study.

In conclusion, our results initially found the upregulated expression of IL-23 and IL-23R *in vitro* or *in situ* ovarian CSLCs. Moreover, autocrine IL-23 could promote self-renewal capacity of CD133^+^ CSLCs *in vitro* and enhanced their tumorigenic potential in xenograft mice via STAT3 and NF-κB signaling. Also, IL-23 levels in primary ovarian cancer specimen are positively correlated with degrees of histological differentiation as well as expression of CSCs-related markers, CD133, Nanog and Oct4. Collectively, our findings provide encouraging evidence of a new therapeutic target to inhibit CSCs-mediated ovarian cancer initiation and progression towards the IL-23/IL-23R/STAT3/NF-κB axis.

## MATERIALS AND METHODS

### Generation and culture of ovarian CD133^+^ CSLCs from cell lines and primary ovarian cancer tissues

This study we use A2780 and SKOV3 cell line as a model system for ovarian tumor. Although A2780 and SKOV3 cells are unknown ovarian cancer subtype, and A2780 and SKOV3 cells does not harbor any mutation or cope number change for TP53 gene, which is a key characteristics of high-grade serous ovarian cancer. These cell lines has been well accepted as a model for high-grade serous adenocarcinoma [55]. The human ovarian cell lines A2780 and SKOV3 were purchased from the American Type Culture Collection (Mannassas, VA, USA). Ovarian CD133+ CSLCs were generated as described previously [24, 25]. A2780 and SKOV3 cells were cultured in DMEM contained 10% fetal bovine serum. CD133+ CSLCs and CD133- cells were purified by FACS (AC133-PE, mouse IgG, Miltenyi) sorting before using. CD133-cells were cultured in RPMI-1640 culture media, containing 10% FBS and 1% penicillin and streptomycin. For stem cells incubation, A2780 and SKOV3 derived ovarian CD133+CSLCs were cultured in serum-free DMEM/F12 (Hyclone, Logan, UT, USA) supplemented with 10 ng/mL of basic fibroblast growth factor (PeproTech Inc., Rocky Hill, NJ, USA), 20 ng/mL of epidermal growth factor (PeproTech Inc.), 5 mg/mL of insulin (Sigma-Aldrich Co. Ltd., St Louis, MO, USA) and 0.4% bovine serum albumin (Sigma). Cell spheres were formed within 3 days after the cells were cultured under these conditions. Then they were mechanically dissociated and re-seeded every 3–4 day intervals. Ovarian cancer tissues were obtained by surgical resection after patient informed consent and approval by the Institutional Review Boards. The patients-derived CD133+CSLCs were isolated by magnetic bead sorting (AC133, mouse IgG, cell isolation kit; Miltenyi, Bergisch Gladbach, Cologne, Germany) using the MidiMACS system as described previously [24].

### Quantitative real-time PCR

Total RNAs were extracted with TRIzol reagent (Invitrogen, Carlsbad, CA, USA). Quantitative PCR was performed to evaluate IL-23 expression in ovarian CSLSs and non-CSLSs. The gene expression was analyzed with a PCR Kit (TaKaRa, Tokyo, Japan), and carried out in triplicate with an ABI 7300 Prism Sequence Detection System (Applied Biosystems, Foster City, CA, USA). The PCR conditions were as follows: 95°C for 30 s, followed by 35 cycles of 95°C for 5s, 60°C for 34s, and 72°C for 45s. The relative gene expression levels were calculated using the comparative Ct (ΔΔCt) method, with GAPDH as a reference gene. The primers used for PCR were as follows: (1) IL-23p19: forward primer: 5′-AGAAGCTCTGCACACTGGC-3′, reverse primer: 5′-CCACACTGGATATGGGGAAC-3′; (2) CD133: forward primer: 5′-CCCGCAGGAGTG AATCTTTTAT-3′,reverse primer: 5′-GAAGTATCTTGA CGCTTTGGTA-3′; (3) Nanog: forward primer: 5′-GTCTCTCCTCTTCCTTCCT-3′, reverse primer: 5′-TTTTTGCGACACTCTTCTC-3′; (4) Oct4: forward primer: 5′-ATGCACAACGAGAGGATTT-3′, reverse primer: 5′-CAGAGTGGTGACGGAGACA-3′; (5) Sox2: forward primer: 5′-AATGCCTTCATGGTGTGG-3′, reverse primer:. 5′-GAGCGTCTTGGTTTTCCG-3′;(6) GAPDH: forward primer: CCACTCCTCCACCTTTGAC, reverse primer: ACCCTGTTGCTGTAGCCA.

### Western blotting

Spheres and cell pellets were collected and lysed in ice-cold NP-40 lysis buffer containing protease inhibitors. The Nuclear-Cytosol Extraction Kit (KeyGen, Nanjing, China) and Phospho-Protein Purification Kit (KeyGen) were used for the extraction of target proteins, respectively. For western blotting analysis, membranes were first labeled with primary antibodies specific for IL-23p19, IL-23p40, IL-23R, p65 (1:100; Santa Cruz Biotechnolgy), Nanog, Oct4, Sox2 (1:500; Abcam), STAT3 and pSTAT3 (1:1000; Cell Signaling Technology). Then the membranes were labeled with HRP-conjugated secondary antibodies (Amersham, Piscat-away, NJ, USA). The signals were visualized by chemiluminescence detection system (chemilmagerTM 5500, Alpha Innotech, San Leandro, CA, USA).

### Flow cytometric analysis

Tumor spheres were dissociated into single cells for flow cytometric analysis, washed and incubated with Percp-conjugated monoclonal antibody (mAb) specific for human IL-23R (IL-23R, mouse IgG2B, R&D Systems) or isotype-matched control mAb (mouse IgG2B, Percp, R&D Systems) for 30 minutes on ice. Cells were washed twice, and IL23R expression was assessed by flow cytometry according to the manufacturer's instructions. The data were analyzed using Flowjo software.

### Immunofluorescence

Samples of spheres and frozen ovarian cancer sections were attached to the cover slips, then fixed in 4% paraformaldehyde solution for 30min and washed three times with PBS. For permeabilization, samples were treated with ice-cold 0.25% Triton X-100 for 30min at room temperature. After permeabilization, samples were washed three times with PBS and blocked with 5% bovine serum albumin in PBS for 30min at room temperature. After washing with PBS, the samples were incubated at 4°C overnight with the following antibodies: mouse monoclonal anti-IL23p19 antibody, goat polyclonal anti-IL23p40 antibody, rabbit polyclonal anti-IL23R antibody (1:50; Santa Cruz Biotechnology), rabbit anti-CD133 antibody, rabbit monoclonal anti-Nanog, mouse anti-Oct4 monoclonal antibody, mouse anti-Sox2 monoclonal antibody (1:200; Abcam) and rabbit anti-p65 monoclonal antibody (1:200; Santa Cruz Biotechnology). Then the samples were washed and incubated at room temperature for 30min with secondary fluorescein (FITC)-conjugated IgG or CY3-conjugated IgG. Nuclei were counterstained with DAPI (40,6-Diamidino-2-Phenylindole, dilactate) after a PBS wash. Cover slips were viewed under fluorescence microscopy (LAS SP5-6000, Wendenstrasse, Hamburg, Germany).

### Sphere formation assay

CD133^+^CSLCs with or without rhIL-23 stimulation were seeded at concentrations of 1, 10, 100 and 1000 cells per well on 96-well plates. Then plates were incubated at 37°C in a humid incubator with 5% CO2 for 2 weeks, spheres containing ≥3 cells were counted under an inverted microscope. During the sphere formation assay, cells were treated with IL-23 neutralizing antibody (R&D Systems), IL-23 inhibitor STA-5326 (Toronto Research Chemicals Inc.), rhIL-23 (R&D Systems), STAT3 inhibitor Cucurbitacin (0.2μM) and p65 inhibitor PDTC (0.5μM).

### RNA interference

IL-23 short hairpin RNA (shRNA) oligonucleotides were purchased from Neuron Bio, Shanghai, China. Briefly, three stem-loop structured oligonucleotides each containing a different IL-23-target sequence were cloned under the control of the human U6 promoter in lentiviral vectors, which also contained a green fluorescent protein (GFP) reporter. The control shRNA had no inserted loop structure. Cells were dissociated into single cells the day before transduction, then transfected with shRNA according to the manufacturer's protocol. After 48 hours, the medium was replaced and cells were collected for further experiments.

### *In vivo* xenograft experiments

Severe combined immunodeficient mice (female, 4–6-week old) were purchased from the Chinese Academy of Medical Sciences (Beijing, China). Mice were maintained and fed in laminar flow cabinets under specific pathogen-free conditions. Then randomly take 26 mice as CD133+ ovarian cancer stem cell group which is transfected by sh-IL-23p19 lentiviral vector or GFP-expressing lentivirus of the 46 female nude mices, and the others are as CD133+ ovarian cancer stem cell group which is stimulated by STA-5326 or DMSO. For xenograft experiments, the above-mentioned two kinds of 5×10^5^ ovarian CD133^+^CSLCs were resuspended in 200 mL of PBS/Matrigel (1:1; BD Biosciences) and injected into the right flank of mice. Engrafted mice were measured biweekly for tumors by visual observation and palpation. At 3-month post transplantation, mice were terminated by cervical dislocation for further evaluation. Mice were cared for and used in accordance with local ethical guidelines.

### Enzyme-linked immunosorbent assay

Supernatants from 1×10^5^ CD133^+^CSLCs and CD133^−^cells from A2780 cells were obtained after 48 hours in culture and stored at −20°C. The concentration of IL-23 in each supernatant was measured using an ELISA kit (TSZ Biotechnology, specific for IL-23p19 subunit) according to the manufacturer's protocol. Absorbance at 450 nm was measured by a microplate reader (Bio-Rad, Hercules, CA, http://www.bio-rad.com). Each measurement was performed in triplicate.

### Statistical analysis

All the data from quantitative assays were expressed as the mean±SD. Statistical analyses were performed using the independent samples t-test or one-way analysis of variance. The difference was considered statistically significant when P<0.05. All statistical analyses were carried out with SPSS 13.0 software (IBM, Armonk, New York, NY, USA).

## SUPPLEMENTARY FIGURES AND TABLE


