# Supplementary material for: Autocrine interleukin-23 promotes self-renewal of CD133+ ovarian cancer stem-like cells
Source: Oncotarget. 2016 Oct 12;7(46):76006–20. doi: 10.18632/oncotarget.12579 (PMC5342794; doi:10.18632/oncotarget.12579)
Supplement: Supplementary file 1 [file oncotarget-07-76006-s001.pdf]

Autocrine interleukin-23 promotes self-renewal of CD133^{+} ovarian cancer stem-like cells

SUPPLEMENTARY FIGURES AND TABLE

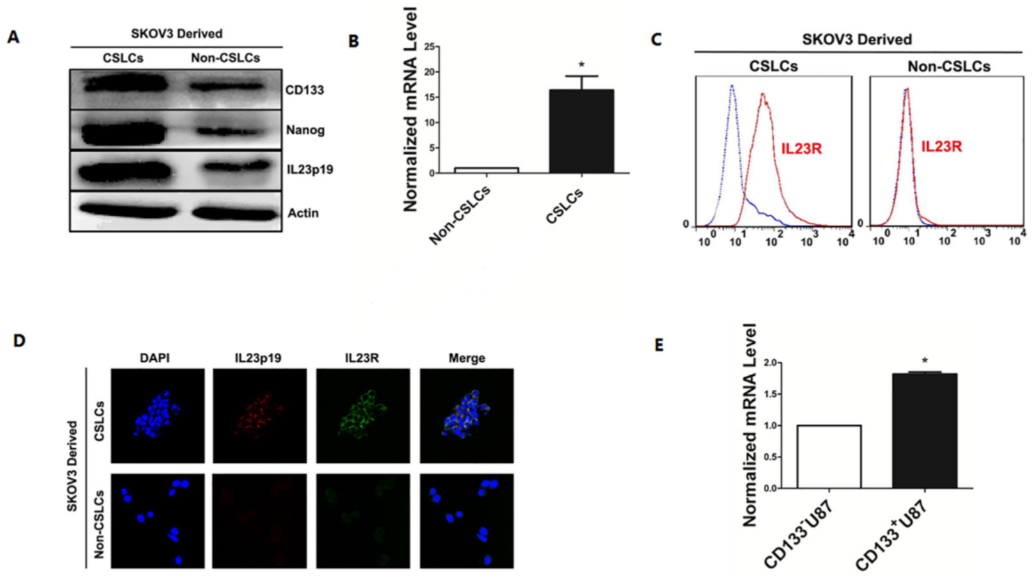

Supplemental Figure S1: IL-23 and IL-23R expression in ovarian CSLCs. A. Western blot analysis of levels of CD133,Nanog and IL-23p19 in CD133^{-} and CD133^{+} cells, derived from SKOV3 cells. **B.** IL-23p19 mRNA expression levels, as measured by real-time PCR, showing an increase in IL-23p19 mRNA production by CD133^{+} cells relative to CD133^{-} cells. **C.** Flow cytometric analysis of IL-23R-expressing cells in SKOV3-derived CD133^{+} and CD133^{-} cells. **D.** Immunofluorescence detection of both IL-23p19 and IL-23R expression in SKOV3-derived CD133^{+} and CD133^{-} cells. **E.** Real-time PCR detection of IL-23p19 mRNA expression levels in U87-derived CD133^{+} and CD133^{-} glioma CSLCs.

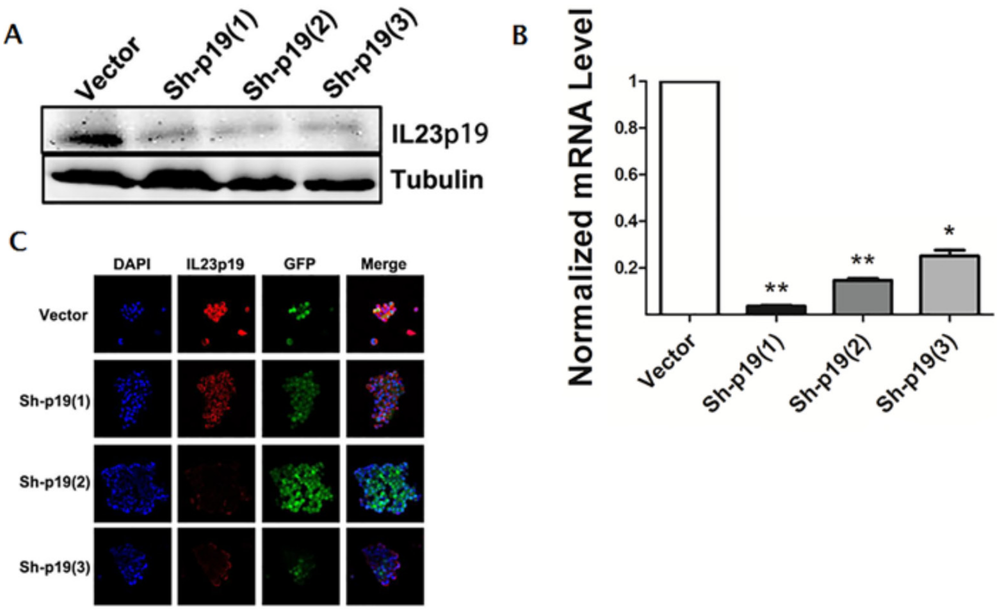

Supplemental Figure S2: The effect of lentivirus-mediated IL-23p19-shRNA on A2780-derived CD133⁺ CSLCs. A. Western blot analysis of levels of IL-23p19 in A2780-derived CD133⁺ CSLCs which were transfected with three kinds of IL-23p19-shRNA. **B.** Real-time PCR analysis of levels of IL-23p19 in A2780-derived CD133⁺ CSLCs which were transfected with three kinds of IL-23p19-shRNA. **C.** Immunofluorescence detection of levels of IL-23p19 in A2780-derived CD133⁺ CSLCs which were transfected with three kinds of IL-23p19-shRNA.

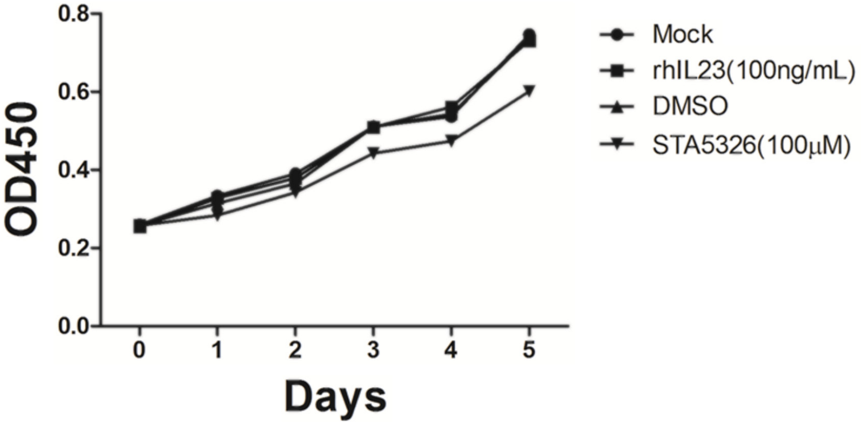

Supplemental Figure S3: The proliferation ability of A2780-derived non-CSLCs. CCK8 proliferation assay analysis of the proliferation ability in A2780-derived non-CSLCs which were treated with rhIL-23 and IL-23 inhibitor STA5326 (100μM).

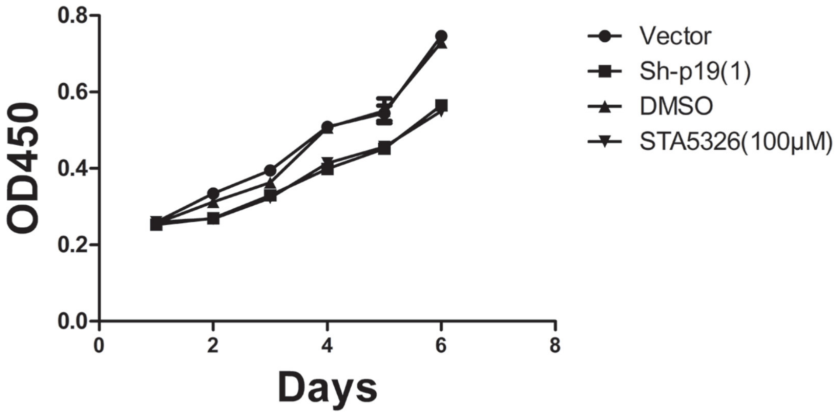

Supplemental Figure S4: The proliferation ability of A2780-derived CD133+ OCSLCs. CCK8 proliferation assay analysis of the proliferation ability in A2780-derived CD133+ OCSLCs which were transfected with the shRNAs directed against IL23-p19 or incubation with STA5326. Data were expressed as means ± S.D. All P values of the data are less than 0.01, with statistical significance.

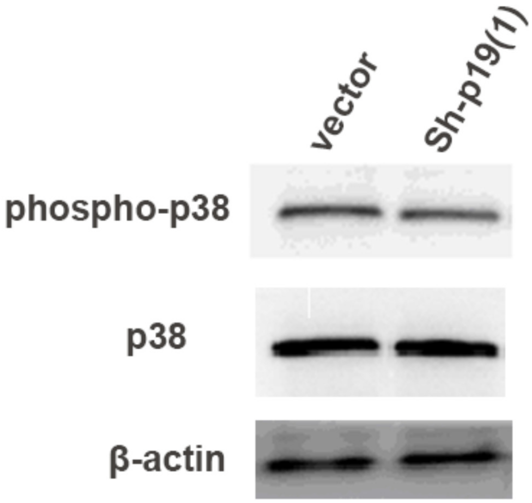

Supplemental Figure S5: The effect of p38MAPK signaling pathways on CD133+ OCSLCs transfected with Control shRNA (vector) or IL-23p19-shRNA. CD133+ OCSLCs were transfected with Control shRNA (vector) or IL-23p19-shRNA, and the phosphorylated and total p38 protein levels were analyzed by Western blot.

Supplemental Table S1: The information of ovarian cancer patients involved in this study

No	Age	Histology	Differentiaiton
1	67	Serous ovarian cancer	Poor
2	51	Serous ovarian cancer	Poor
3	52	Serous ovarian cancer	Well
4	49	Serous ovarian cancer	Poor
5	43	Serous ovarian cancer	Well
6	45	Serous ovarian cancer	Well
7	56	Serous ovarian cancer	Poor
8	41	Serous ovarian cancer	Poor
